# Structure and diversity of microbial communities in the rhizosphere soil of *Trichosanthes kirilowii* from different cultivation patterns

**DOI:** 10.7717/peerj.20459

**Published:** 2025-11-27

**Authors:** Lei Zheng, Huadong Wang, Zhiqiang Zhang, Jiulin Gu, Yao Yin

**Affiliations:** 1 School of Pharmacy, Sichuan College of Traditional Chinese Medicine, Mianyang, Sichuan, China; 2 Northwest Sichuan Laboratory of Traditional Chinese Medicine Resources Research and Development Utilization, Mianyang, Sichuan, China; 3 Mianyang Key Laboratory of Development and Utilization of Chinese Medicine Resources, Mianyang, Sichuan, China

**Keywords:** *Trichosanthes kirilowii*, Bacterial community, Fungal community, Community structure, Diversity, Open-field cultivation, Film-mulched cultivation, Soybean intercropping cultivation

## Abstract

**Background:**

To analyze the effects of different cultivation patterns on the structure and diversity of the microbial community in the rhizosphere soil of *Trichosanthes kirilowii* (*T. kirilowii*) arms to establish reasonable and effective strategies to mitigate the continuous cropping barriers and promote the high-quality cultivation of *T. kirilowii*.

**Methods:**

Three distinct cultivation patterns were investigated: open-field cultivation (TM1), film-mulched cultivation (TM2), and soybean intercropping cultivation (TM3). High-throughput sequencing and bioinformatic analyses were employed to evaluate the rhizosphere microbiome, and redundancy analysis was utilized to investigate the relationship between the microbial communities and soil nutrient indicators.

**Results:**

TM2 and TM3 increased soil bacterial community diversity, reduced fungal community diversity, elevated the relative abundance of beneficial bacterial genera, and reduced the abundance of detrimental fungal genera in the rhizosphere soil. The relative abundance of *Pseudarthrobacter*, *unclassified_Steroidobacteraceae*, and *Nocardioides* in TM2 and TM3 was markedly higher than in TM1. Conversely, the relative abundance of *Fusarium*, *Rhizoctonia*, *Ceratobasidium*, and *Plectosphaerella* in TM2 and TM3 was significantly reduced compared to TM1. The contents of available potassium (AK), total nitrogen (TN), total phosphorus (TP), and pH in the rhizosphere soil of TM2 and TM3 were significantly higher than those in TM1. The distribution of soil bacterial genera was significantly influenced by the contents of TN and AK, while the distribution of soil fungal genera was significantly or extremely significantly impacted by the contents of TP, total potassium (TK), soil organic matter (SOM), and pH. The content of AK was extremely significantly positively correlated with the relative abundance of *Nocardioides*, whereas the content of TK showed an extremely negative correlation with the relative abundance of *Ceratobasidium*. Similarly, pH demonstrated an extremely negative correlation with the relative abundance of *Rhizoctonia* and *Ceratobasidium*.

**Conclusions:**

Film-mulched cultivation and soybean intercropping cultivation altered the soil nutrients, as well as the structure and diversity of soil microbial communities. Thus, in agricultural production, film-mulched cultivation and soybean intercropping cultivation can serve to regulate soil nutrients and microbial communities, thereby mitigating the barriers of continuous cropping of *T. kirilowii*.

## Introduction

The perennial medicinal plant *Trichosanthes kirilowii* Maxim., a member of the Cucurbitaceae family, its fruits, seeds, peels, and roots that are all used in traditional Chinese medicine, known respectively as Gualou, Gualou seeds, Gualou peel, and Tianhua fen (National Pharmacopoeia Commission, 2020). *T. kirilowii* is distributed in China (Anhui, Sichuan, Shandong, Henan, Hebei, and Jiangsu provinces), Japan, and South Korea and is classified as one of the fifty most important medicinal plants used in traditional Chinese medicine (Leung et al., 2020; Lewtak et al., 2024). Notably, there are about 162 chemicals that have been isolated and identified from *T. kirilowii*, including terpenoids, phytosterols, flavonoids, nitrogenous compounds, and lignans. Several investigations have demonstrated the pharmacological properties of the extracted extracts and chemicals, which include antioxidant, anti-hypoxic, anti-platelet aggregation, expectorant, anti-inflammatory, and cytotoxic properties, and protection against myocardial ischemia (Yu et al., 2018; Xie et al., 2020). Currently, *T. kirilowii* is commonly used to treat angina, thoracic obstruction, constipation, and pulmonary heart disease and is widely applied in the pharmaceutical, food, and cosmetics industries, demonstrating considerable medicinal, edible, and economic value (National Pharmacopoeia Commission, 2020; Yang et al., 2019). *T. kirilowii* is a climbing crop that requires support structures to ensure normal growth during cultivation, resulting in high production costs (Joo et al., 2019). As a result, *T. kirilowii* cultivation typically adopts an open-field cultivation pattern, leading to issues with continuous cropping barriers, soil degradation, growth inhibition, and increased pest and disease incidence, ultimately resulting in diminished yield and quality (Chen et al., 2023; He et al., 2025; Zhang et al., 2022).

Long-term continuous cropping led to a significant decrease in soil organic carbon, total nitrogen, alkali-hydrolyzable nitrogen, and available phosphorus contents as well as soil enzyme activities, which resulted in accelerated nutrient consumption, nutrient imbalances, and deteriorated soil physicochemical properties (Wu et al., 2022; Li et al., 2024b). Due to the soil degradation caused by long-term continuous cropping, which significantly affected the structure and diversity of microbial communities, it disrupted the equilibrium of soil microorganisms, inhibiting the survival of beneficial microbes while promoting the proliferation of pathogenic ones, thereby intensifying the incidence of soil-borne diseases, ultimately impeding plant growth and development (Liu et al., 2021a; Ding et al., 2021). Soil microorganisms are integral to the soil ecosystem (Peddle et al., 2025), contributing to processes such as soil organic matter decomposition (Shahbaz et al., 2017) and nutrient cycling (Wang et al., 2024). They are instrumental in facilitating plant growth, bolstering plant resistance, and sustaining soil ecological health (El Bey et al., 2025). Studies indicated that the soil bacterial community structures of tomatoes, tobacco, and sweet potatoes were significantly influenced by continuous cropping barriers and were notably affected by their duration (Gao et al., 2021; Jiang et al., 2024; Li et al., 2024a). Deng et al. (2023) found that as the years of continuous cropping increase, the relative abundance of beneficial microorganisms in rice/cherry tomato soil decreases, while the relative abundance of pathogenic fungi increases, leading to alterations in the microbial community structure, which negatively affects growth and development. Li et al. (2022a) demonstrated that long-term continuous cropping of lilies alters soil microbial quantity and community structure; the Shannon and Simpson diversity indexes were significantly increased for bacteria and significantly decreased for fungi. Meanwhile, *Fusarium* dominated in the Ascomycota after continuous cropping, which might be one of the main reasons for the continuous cropping obstacle in *lily*. Kang et al. (2018) confirmed that long-term continuous cropping of tomatoes reduced soil microbial abundance, with microbial community structure showing significant changes with increasing cropping duration. Zhou et al. (2022) investigated the dynamic changes in rhizosphere bacterial communities of *Fritillaria taipaiensis* across varying planting years, finding that the Shannon and Simpson indices of rhizosphere soil bacterial communities decreased with increasing planting years.

Changes in the structure and diversity of rhizosphere microbial communities are influenced not only by cultivation management systems, crop type, soil type, and nutrients but also by cultivation patterns, which all lead to variations in root exudates, ultimately affecting microbial community structure and diversity (Pu et al., 2024; Song et al., 2023; Tong et al., 2021; Anderson et al., 2024; Steinauer, Chatzinotas & Eisenhauer, 2016). Different cultivation patterns can effectively adjust soil properties and improve the soil’s microenvironment, thereby creating a conducive rhizosphere growth environment and promoting plant growth and development (Zhang et al., 2025a). Tian et al. (2023) discovered that different cultivation patterns significantly influence the Chao and ACE indices of fungal communities and soil properties in watermelon cultivation. Zhang et al. (2025b) analyzed the differences in the quality of *Epimedium koreanum Nakai* and soil microhabitats under different cultivation methods, demonstrating that the physicochemical properties and enzyme activity of the soil under different cultivation methods affect soil microbial diversity and composition, thereby influencing plant growth and the synthesis of key components. The structure and diversity of soil microbial communities in the *ginseng* rhizosphere were influenced by cultivation patterns; increasing the ages of *P. ginseng* cultivation led to a decrease in bacterial diversity and an increase in fungal diversity, while the richness and diversity of fungal communities were higher in forests than in farmland, and the bacterial communities had lower values in forests than in farms (Xiao et al., 2016; He et al., 2022). Chen et al. (2025b) found that crop rotation alleviated continuous cropping obstacles in *Chrysanthemum morifolium* production by regulating rhizosphere soil microbial communities and metabolites, whose soil nutrient enrichment and enzymatic activity enhancement in the rotation system were primarily influenced by Actinobacteria, Cyanobacteria, unclassified bacteria, and Basidiomycota. Film-mulched cultivation and intercropping cultivation are commonly used in China’s crop cultivation. Studies have indicated that film-mulched cultivation and intercropping cultivation can alter the community structure and diversity of rhizosphere microbes, thereby increasing crop yield (Dang et al., 2020; Kang et al., 2020; Li et al., 2022b). Due to the large demand in the marketplace, there have been established many bases for *T. kirilowii* in China. However, the cultivation of *T. kirilowii* is attacked by continuous cropping barriers, leading to substantial yield losses, posing a serious threat to the *T. kirilowii* industry. Therefore, it is imperative to explore effective methods that can mitigate these obstacles. Despite the extensive research and application of film-mulched cultivation and intercropping cultivation, their impact on the rhizosphere microbial community of *T. kirilowii* remains largely unexplored. This study focuses on *T. kirilowii* subjected to different cultivation patterns, employing high-throughput sequencing technology to analyze the structure and diversity of the rhizosphere microbial communities and elucidating the relationship between these microbes and soil nutrient indicators. The objective is to gain a better understanding of the previously unknown mechanistic link between cultivation patterns and the rhizosphere soil microbiome of *T. kirilowii* in order to develop effective strategies for overcoming continuous cropping barriers, thereby providing a scientific foundation for promoting high-quality *T. kirilowii* cultivation.

## Materials and Methods

### Experimental site and overview

The experimental site is located in Baotu Village, Chengjiao Township, Fucheng District, Mianyang City, Sichuan Province, at 104°41′6.576″E, 31°30′28.865″N. The soil type is clay soil and elevation is 489 m. The pH fluctuates from 7.0 to 9.0, the organic matter content ranges from 15 to 25 g/kg, and the available potassium content ranges from 80 to 150 mg/kg. The area has a subtropical humid monsoon climate, with an annual average temperature of 16.3 °C, an annual average sunshine duration of 1,298.1 h, a frost-free period of 272 days, and an annual average precipitation of 963.2 mm (Jiang et al., 2025).

### Experimental design

The experiment employed a randomized block design, incorporating three distinct planting patterns: open-field cultivation (TM1), film-mulched cultivation (TM2), and soybean intercropping cultivation (TM3). Each pattern was replicated thrice, resulting in nine plots, each with an area of 15 m × 2 m = 30 m^2^. In each plot, 10 *T. kirilowii* plants were planted, using their tubers for sowing, which were sourced from Anhui Province, China. In the open-field and film-mulched cultivation plots, single-row planting was used with a spacing of 1.5 m × 2.0 m of *T. kirilowii*. In the *T. kirilowii*-soybean intercropping cultivation, the layout included a single row of *T. kirilowii* accompanied by four rows of soybeans. The spacing for *T. kirilowii* remained at 1.5 m × 2.0 m, while soybeans were positioned with a spacing of 10 cm × 40 cm. Soybeans were sown using seeds, and the variety was Jiaoda No. 18 (Shandong Shouhe Seeds Industry Co., Ltd., Shandong, China). *T. kirilowii* was planted in March 2024, with soybeans sown in April 2024. Field operations were managed conventionally after sowing, with trellising during the vine-stretching stage, with topdressing applied during the seedling stage, flowering stage, and fruit ripening stage. Flood irrigation was employed during drought periods.

### Sample collection

During the fruit mature phase of *T. kirilowii*, a five-point sampling method was utilized within each plot to randomly procure rhizosphere soil from five plants. A sterile metal spatula was used to dig the soil profile at 0–30 cm depth, collecting the *T. kirilowii* rhizosphere soil that was not removed after shaking, and then transferred it into sterile sample bags for further processing. The rhizosphere soil collected from each plot was mixed to form a single composite sample for testing. Three such samples were obtained for each cultivation pattern, yielding a total of nine samples. Each soil sample was divided into two parts, one for soil microbial analysis and the other for soil nutrient assessment.

### Determination of rhizosphere soil nutrient indicators

The rhizosphere soil nutrient indicators of *T. kirilowii* were determined using the method of Bao Shidan (BAO, 2000). The total nitrogen (TN) content in soil was determined using the Kjeldahl method, and alkali-hydrolyzed nitrogen (AN) content was quantified using the alkali-hydrolyzed diffusion method. The total phosphorus (TP) and available phosphorus (AP) content were measured using the sodium hydroxide alkali fusion-molybdenum antimony colorimetric method, the total potassium (TK) and available potassium (AK) content were evaluated using the flame photometric method, the soil organic matter (SOM) content was determined using the potassium dichromate volumetric method, and the soil pH was gauged using the potentiometric method. Each soil nutrient indicator was measured in triplicate.

### DNA abstraction, PCR enlargement, and High-throughput sequencing

Soil total DNA was extracted using the TGuide S96 kit (TianGen Biotechnology (Beijing) Co., Ltd., model: DP812). Three samples per cultivation pattern were analyzed. DNA concentration was measured using the Synergy HTX, and integrity was assessed *via* agarose gel electrophoresis at a concentration of 1.8%. The primers 338F (5′-ACTCCTACGGGAGGCAGCA-3′) and 806R (5′-GGACTACHVGGGTWTCTAAT-3′) were adopted for the amplification of the V3–V4 hypervariant areas for the bacterium 16S rRNA gene (Liu et al., 2016). ITS1F (5′-CTTGGTCATTTAGAGGAAGTAA-3′) and ITS2R (5′-TCCTCCGCTTATTGATATGC-3′) were selected to target the ITS1-ITS2 region for the characterization of fungal communities (Zhang et al., 2024b). The PCR reaction system of 10 μL consisted of genomic DNA (2.5–4 ng), Vn F (10 μmol·L^−1^) 0.3 μL, Vn R (10 μmol·L^−1^) 0.3 μL, KOD FX Neo Buffer 5 μL, dNTP (2 mmol·L^−1^ each) 2 μL, KOD FX Neo 0.2 μL, and ddH_2_O to make up to 10 μL. PCR amplification conditions included 95 °C for 5 min, followed by 27 cycles at 95 °C denaturation for 30 s, 55 °C annealing for 30 s, and 72 °C extension for 30 s, and a final elongation at 72 °C for 7 min. 1.8% agarose gel was used to confirm the size of the amplified product, which was then sequenced on the Illumina Novaseq6000 by Beijing Qingke Biotechnology Co., Ltd. Read length was 250 bp at both ends, with a sequencing depth exceeding 50,000 reads per sample.

### Bioinformatics analysis

Primer and adapter sequences were trimmed using Cutadapt (Martin, 2011). Subsequent quality filtering was performed with Trimmomatic to remove low-quality bases (Phred score < 20) and short reads (<200 bp) (Bolger, Lohse & Usadel, 2014). The resulting high-quality paired-end reads were assembled into full-length amplicon sequences using FLASH 1.2.7 (Magoc & Salzberg, 2011). Chimeric sequences were identified and removed using UCHIME (Edgar et al., 2011). Operational taxonomic units (OTUs) were clustered from the high-quality sequences using the UPARSE 7.1 algorithm at a 97% similarity threshold (Edgar, 2013). The bacterium 16S rRNA alignment database was Silva 138 (Quast et al., 2013), and the ITS fungal alignment database was Unite 8.0 (Koljalg et al., 2005). The taxonomic-level community structure map was derived from the OTU analysis. Calculations for Chao 1, Shannon, and Simpson indices were conducted employing mothur software (Schloss et al., 2009). Principal coordinate analysis (PCA) based on Bray-Curtis dissimilarity was performed to assess the similarity of microbial community structures. Linear discriminant analysis effect size (LEfSe) using the Galaxy online analytics platform to perform. Redundancy analysis (RDA) was used to investigate the relationship between the relative abundance of microbial communities at the genus level in rhizosphere soil and soil nutrients. Additionally, the cloud platform tool from Beijing Qingke Biological Technology Co., Ltd. was utilized for graphing.

### Statistical analysis

Data were managed using Excel 2016, while statistical analyses were performed with SPSS Statistics 19 (Chicago, IL, USA). The one-way ANOVA and the Duncan’s (α = 0.05) test were adopted to contrast the soil nutrient indicators and microbial level and diversity (Jiao et al., 2022). Spearman’s correlative was adopted for the establishment of an association among microbe genera and soil nutrient indicators.

### Sequence registration numbers

The sequence data were deposited in the NCBI Sequence Read Archive (SRA) database with the accession number of SRR35124295–SRR35124303.

## Results

### Effects of different cultivation patterns on rhizosphere soil nutrients of *T. kirilowii*

Differences in rhizosphere soil nutrients were observed among different cultivation patterns (Table 1). The content of AK, TN, TK, and pH value in the rhizosphere soil of TM2 and TM3 was significantly higher than that of TM1 (*P* < 0.05). However, there were no significant differences in soil AN content across the three patterns. SOM content in TM3 was significantly higher than in TM1, while TP content was significantly lower than in TM1 (*P* < 0.05).

**Table 1:** Nutrient indicators in the rhizosphere soil of *T. kirilowii* from different cultivation patterns.

Pattern	SOM (g/kg)	AN (mg/kg)	AP (mg/kg)	AK (mg/kg)	TN (g/kg)	TP (g/kg)	TK (g/kg)	pH
TM1	20.32 ± 0.28 b	41.97 ± 0.14 a	26.47 ± 0.14 b	109.72 ± 5.48 b	0.65 ± 0.01 c	1.10 ± 0.01 a	11.64 ± 0.08 c	8.56 ± 0.01 c
TM2	20.44 ± 0.24 b	41.30 ± 0.29 a	37.49 ± 0.24 a	121.58 ± 3.69 a	0.71 ± 0.01 a	1.12 ± 0.01 a	12.28 ± 0.03 b	8.61 ± 0.01 b
TM3	21.51 ± 0.24 a	42.39 ± 0.25 a	24.18 ± 0.13 c	120.60 ± 2.27 a	0.68 ± 0.01 b	0.94 ± 0.01 b	14.13 ± 0.09 a	8.71 ± 0.01 a

**Notes:**

Different letters indicate significant differences at *P* < 0.05. Data are presented as the mean ± standard deviation (*n* = 3).

TM1, open-field cultivation; TM2, film-mulched cultivation; TM3, soybean intercropping cultivation; SOM, organic matter; AN, available nitrogen; AP, available phosphorus; AK, available potassium; TN, total nitrogen; TP, total phosphorus; TK, total potassium.

### Sequence data of microbial communities in the rhizosphere soil of *T. kirilowii*

**Figure 1:**
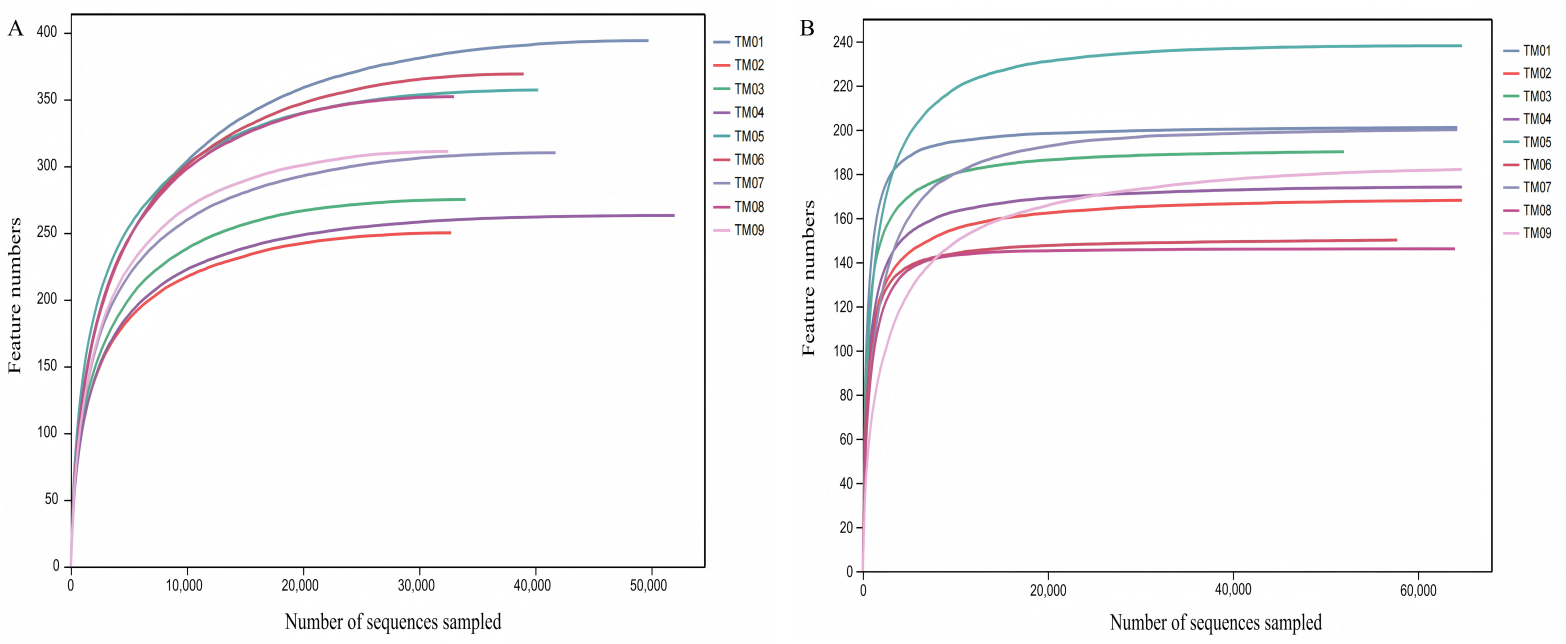
Dilution curves of bacterial and fungal communities of *T. kirilowii*. (A) Bacteria. (B) Fungi. TM01-TM03 are samples from open-field cultivation, TM04-TM06 are samples from film-mulched cultivation, and TM07–TM09 are samples from soybean intercropping cultivation.

High-throughput sequencing was performed from nine soil samples of 3 *T. kirilowii* cultivation patterns, yielding a total of 753,832 bacterial raw sequences, with 672,057 valid sequences after quality control; similarly, 938,504 fungal raw sequences were obtained, with 811,071 valid sequences post-quality control. Dilution curve analysis based on randomly selected sequence depth and species quantity (Fig. 1) showed that the dilution curves of soil sample sequences progressively flattened as the bacterial and fungal sequencing reached 30,000 and 20,000 reads, respectively, indicating that the sequencing results encompassed the majority of bacterial and fungal types in the samples, effectively reflecting the community composition of bacteria and fungi in the soil.

**Figure 2:**
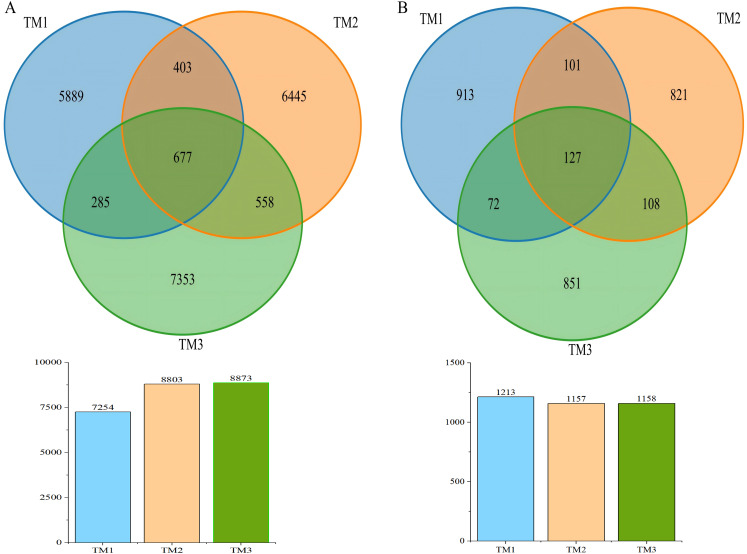
Venn diagram of OTU numbers in bacteria and fungi of *T. kirilowii* from different cultivation patterns. (A) Bacteria. (B) Fungi. TM1, open-field cultivation; TM2, film-mulched cultivation; TM3, soybean intercropping cultivation.

A Venn diagram was used to visualize the Operational Taxonomic Units (OTUs) in soil samples from the three cultivation patterns (Fig. 2). The results showed that the bacterial OTUs in the TM1, TM2, and TM3 soil samples were 7,254, 8,083, and 8,873, respectively. Notably, 677 bacterial OTUs were common to all soil samples, while 5,889, 6,445, and 7,353 bacterial OTUs were unique to the TM1, TM2, and TM3 soil samples, respectively; as for fungal OTUs, the TM1, TM2, and TM3 soil samples were 1,213, 1,157, and 1,158, respectively. Among these, 127 fungal OTUs were common to all soil samples, while 913, 821, and 851 fungal OTUs were unique to the TM1, TM2, and TM3 soil samples, respectively.

### Analysis of $ \alpha$-diversity in the rhizosphere soil microbial communities

**Figure 3:**
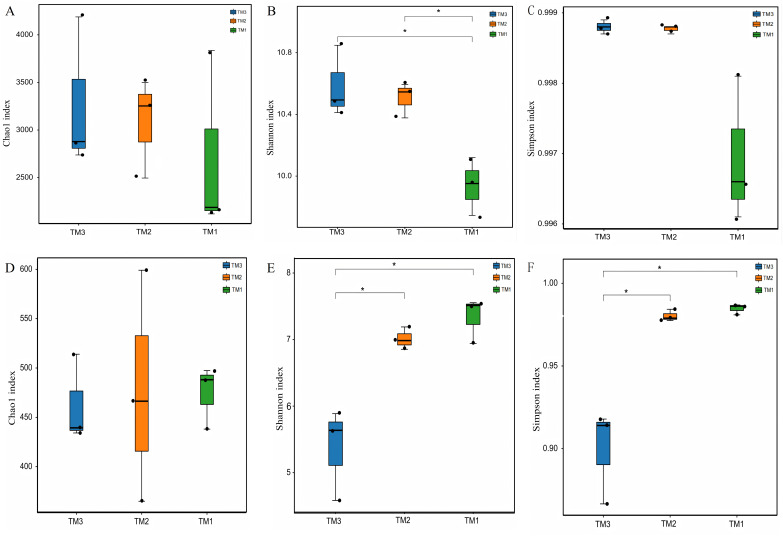
$ \alpha$-diversity of rhizosphere soil microbial communities of *T. kirilowii* from different cultivation patterns. (A–C) Bacteria. (D–F) Fungi. TM1, open-field cultivation; TM2, film-mulched cultivation; TM3, soybean intercropping cultivation. *, represents *P* < 0.05.

To compare the α-diversity of microbial communities, the Chao1 index was employed to represent community abundance, while the Shannon and Simpson indices were utilized to denote community diversity. The effects of cultivation patterns on soil microbial communities’ abundance and diversity were analyzed. The results of bacterial community abundance and diversity (Figs. 3A–3C) revealed that the Chao1 index values for TM1, TM2, and TM3 soil samples were 2,713.6, 3,082.1, and 3,268.1, respectively, with no significant differences among the three cultivation patterns; however, the Shannon indices for TM2 and TM3 soil samples were 10.6 and 10.4, both notably higher than the 9.7 for TM1. The Simpson indices of bacterial communities in TM1, TM2, and TM3 soil samples were 0.9988, 0.9988, and 0.9969, respectively, with no significant differences among the three cultivation patterns. In contrast, the results of fungal community abundance and diversity analysis (Figs. 3D–3F) showed that Chao1 index values for TM1, TM2, and TM3 were 474.5256, 476.8145, and 462.5065, respectively, with no significant disparities found among the three cultivation patterns; however, the Shannon and Simpson indices for TM1 and TM2 were 7.3364 and 0.9847, and 7.0095 and 0.9802, respectively, which were significantly elevated compared to the 5.3686 and 0.8995 for TM3.

### Analysis of $ \beta$-diversity in the rhizosphere soil microbial communities

**Figure 4:**
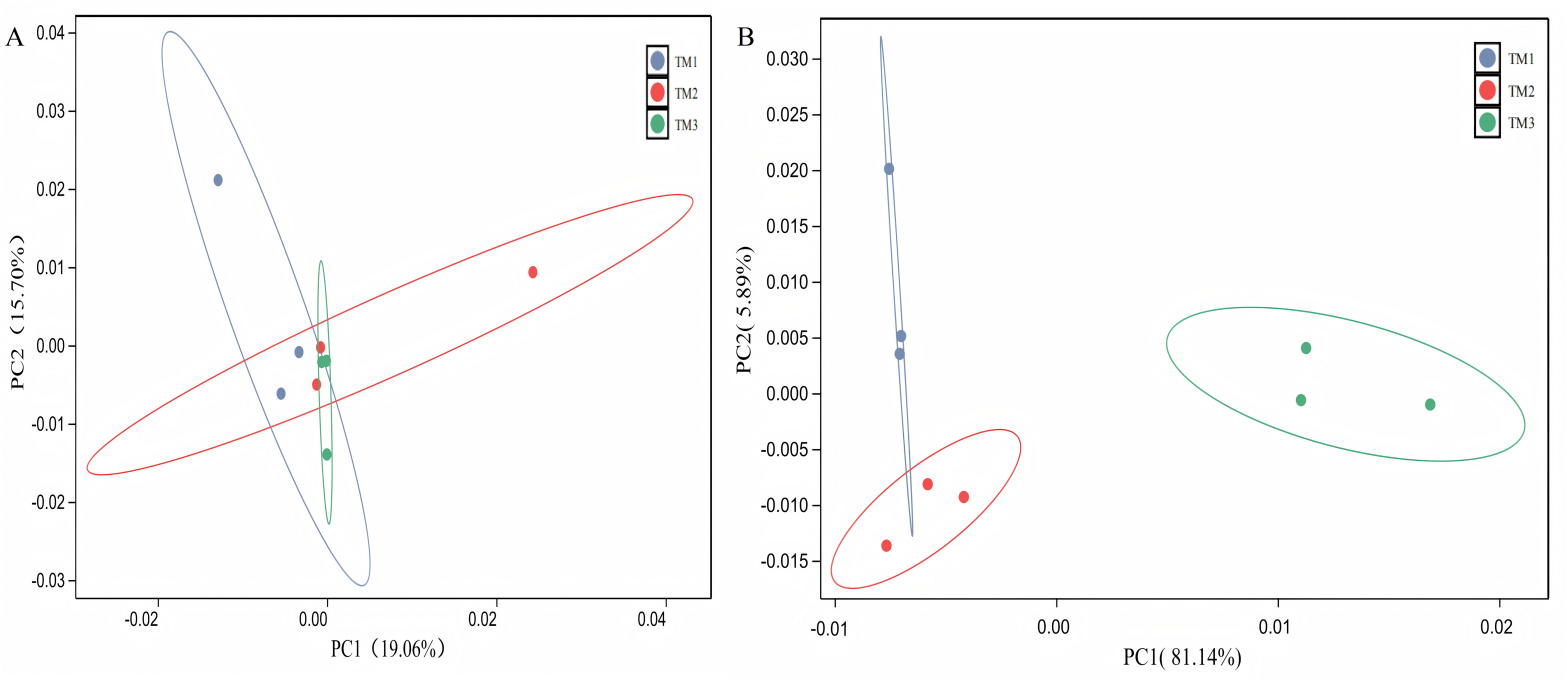
Principal component analysis of rhizosphere soil microbial communities of *T. kirilowii* from different cultivation patterns. (A) Bacteria. (B) Fungi. TM1, open-field cultivation; TM2, film-mulched cultivation; TM3, soybean intercropping cultivation.

The PCA of bacterial communities revealed that PC1 and PC2 contributed 19.06% and 15.70%, respectively, to the variance. The bacterial community structure in TM1 was distinct from that of TM2 and TM3; however, the dispersion of TM1, TM2, and TM3 soil samples was small, with overlap between samples, indicating that film-mulched cultivation and soybean intercropping cultivation had a minor impact on the bacterial community structure in the rhizosphere soil of *T. kirilowii* (Fig. 4A). For fungal communities, PCA results indicated that PC1 and PC2 contributed 81.140% and 5.89%, respectively. The fungal community structure in TM3 was notably different from that of TM2 and TM1, with TM3 soil samples being farther from TM2 and TM1, signifying that soybean intercropping cultivation has a significant impact on the structure of fungal communities. Conversely, the fungal community structure of TM2 and TM1 intersected, indicating that film-mulched cultivation has a relatively minor effect on soil fungal community structure (Fig. 4B).

### Analysis of microbial community structure at the phylum level

**Figure 5:**
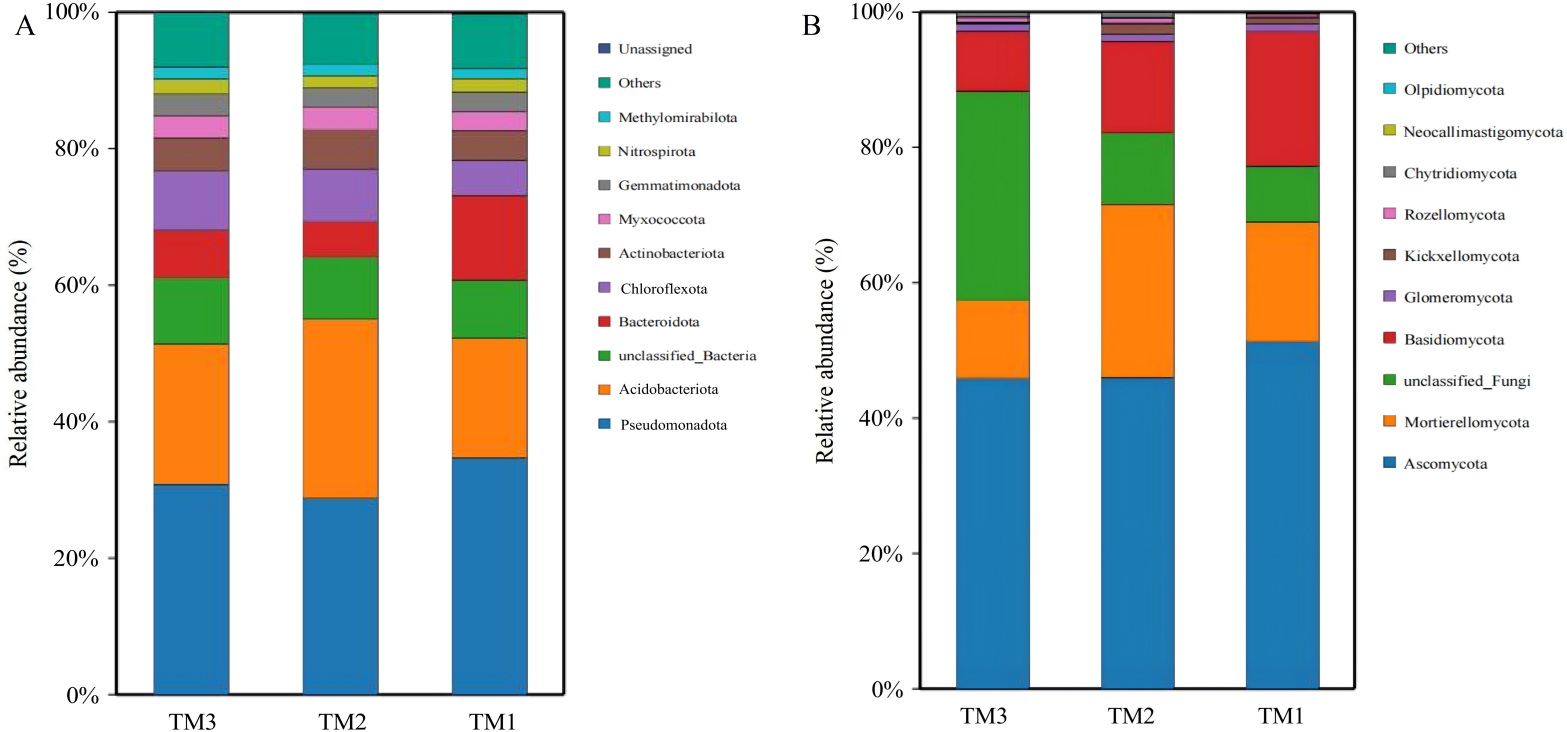
Community structure of bacterial and fungal phyla of *T. kirilowii* from different cultivation patterns. (A) Bacteria. (B) Fungi. TM1, open-field cultivation; TM2, film-mulched cultivation; TM3, soybean intercropping cultivation.

Bacteria in soil samples from the three cultivation patterns were classified into 40 phyla, and fungi were classified into 13 phyla. Among the 40 phyla of bacterial communities from the three cultivation patterns, the dominant phyla with relative abundances exceeding 5.0% were Pseudomonadota (28.8–34.7%), Acidobacteriota (17.5–20.6%), unclassified_Bacteria (8.5–9.7%), Bacteroidota (5.2–12.4%), and Chloroflexota (5.1–8.7%). By comparing the relative abundances of dominant bacterial phyla (Fig. 5A), it was revealed that Acidobacteriota had a relative abundance of 17.5% in TM1, increasing by 8.8% and 3.1% in TM2 and TM3, respectively; Pseudomonadota had a relative abundance of 34.7% in TM1, decreasing by 5.9% and 3.9% in TM2 and TM3, respectively. Furthermore, the results of the relative abundance of bacterial phyla with important functions from different cultivation patterns (Table 2) indicate that the relative abundance of Bacteroidota in TM2 and TM3 was notably higher than in TM1, increasing by 2.47% and 1.71%, respectively, compared to TM1; the relative abundance of Chloroflexota in TM3 also significantly surpassed that in TM1 by 4.23%. However, the relative abundance of Nitrospirota across the three cultivation patterns remained statistically insignificant.

**Table 2:** Relative abundance of some important functional bacterial phyla from different cultivation patterns.

Phylun	TM1	TM2	TM3
Bacteroidota (%)	5.33 ± 0.77 b	7.80 ± 0.25 a	7.04 ± 0.28 a
Chloroflexi (%)	4.36 ± 0.54 b	5.76 ± 1.40 b	8.59 ± 1.44 a
Nitrospirota (%)	2.02 ± 0.46 a	1.86 ± 0.20 a	2.21 ± 0.02 a

**Notes:**

Different letters indicate significant differences at *P* < 0.05. Data are presented as the mean ± standard deviation (*n* = 3).

TM1, open-field cultivation; TM2, film-mulched cultivation; TM3, soybean intercropping cultivation.

Among the 13 phyla of fungal communities in soil samples from the three cultivation patterns (Fig. 5B), the dominant phyla with relative abundances exceeding 5.0% were Ascomycota (45.9–51.3%), Mortierellomycota (11.5–25.6%), unclassified_Fungi (8.3–30.9%), and Basidiomycota (9.0–19.8%). Compared to TM1, the relative abundance of Mortierellomycota increased by 8.0% in TM2, while the relative abundances of Basidiomycota and Ascomycota decreased by 6.3% and 5.3%, respectively. TM3 demonstrated a notable 22.6% rise in the relative abundance of unclassified_Fungi compared to TM1, while the relative abundances of Acidobacteriota, Mortierellomycota, and Basidiomycota decreased by 5.4%, 6.2%, and 10.9%, respectively.

### Analysis of microbial community structure at the genus level

**Figure 6:**
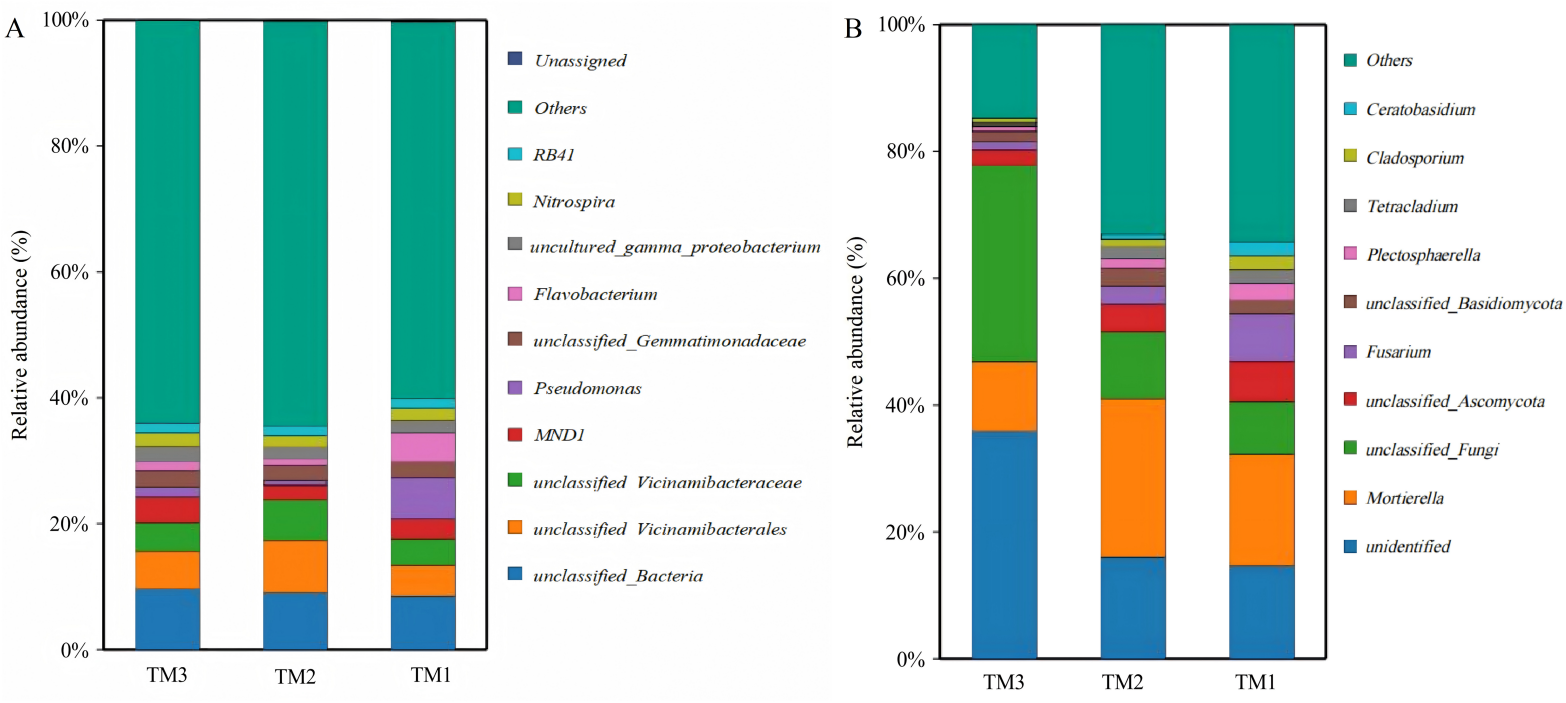
Community structure of bacterial and fungal genus of *T. kirilowii* from different cultivation patterns. (A) Bacteria. (B) Fungi. TM1, open-field cultivation; TM2, film-mulched cultivation; TM3, soybean intercropping cultivation.

A total of 1,204 bacterial genera and 439 fungal genera were identified across all soil samples from the three different cultivation patterns. An analysis was conducted on the dominant bacterial genera with a relative abundance greater than 1.50% among all 1,204 bacterial genera (Fig. 6A). The results showed that there were 11 dominant bacterial genera with relative abundances greater than 1.5% in TM1, including *unclassified_Bacteria* (8.6%), *Pseudomonas* (6.3%), *unclassified_Vicinamibacterales* (5.1%), *Flavobacterium* (4.3%), *unclassified_Vicinamibacteraceae* (4.2%), *MND1* (3.2%), *unclassified_Gemmatimonadaceae* (2.5%), *uncultured_gamma_proteobacterium* (2.0%), *Nitrospira* (2.0%), *Chryseobacterium* (1.7%), and *RB41* (1.5%). In comparison to TM1, TM2 introduced a new dominant genus, *unclassified_Subgroup_17* (1.7%), while increasing the relative abundances of *unclassified_Vicinamibacterales* and *unclassified_Vicinamibacteraceae* to 8.3% and 6.3%, respectively. In TM3, the dominant bacterial genera newly added were *unclassified_A4b* (1.7%), *unclassified_Subgroup_17* (1.6%), and *unclassified_Alphaproteobacteria* (1.5%), while the relative abundance of *Pseudomonas* decreased to 1.5%. In addition to dominant bacterial genera, there were also significant differences in some potentially beneficial bacterial genera among the three cultivation patterns (Table 3). Compared with TM1, the relative abundances of potential beneficial bacterial genera *Nitrosospira*, *Bryobacter*, *Pseudarthrobacter*, *unclassified_Steroidobacteraceae*, *Nocardioides*, and *Agromyces* in TM2 increased by 1.7-fold, 2.0-fold, 1.3-fold, 1.5-fold, 2.0-fold, and 1.5-fold, respectively (*P* < 0.05). In TM3, the relative abundances of *Pseudarthrobacter*, *unclassified_Steroidobacteraceae*, and *Nocardioides* increased by 1.4-fold, 1.4-fold, and 1.6-fold, respectively, compared to TM1 (*P* < 0.05). The above analysis results indicate that there are significant differences in the bacterial community structure of the *T. kirilowii* rhizosphere soil from different cultivation patterns.

**Table 3:** Relative abundance of some beneficial bacterial genera from different cultivation patterns.

Genus	TM1	TM2	TM3
*Nitrosospira* (%)	0.038 ± 0.003 b	0.063 ± 0.001 a	0.049 ± 0.012 ab
*Bryobacter* (%)	0.295 ± 0.031 b	0.595 ± 0.164 a	0.414 ± 0.034 ab
*Pseudarthrobacter* (%)	0.596 ± 0.069 b	0.745 ± 0.048 a	0.814 ± 0.032 a
*Unclassified_Steroidobacteraceae* (%)	0.306 ± 0.037 b	0.457 ± 0.039 a	0.430 ± 0.064 a
*Nocardioides* (%)	0.292 ± 0.029 b	0.584 ± 0.110 a	0.459 ± 0.073 a
*Agromyces* (%)	0.130 ± 0.027 b	0.199 ± 0.013 a	0.146 ± 0.012 b

**Notes:**

Different letters indicate significant differences at *P* < 0.05. Data are presented as the mean ± standard deviation (*n* = 3).

TM1, open-field cultivation; TM2, film-mulched cultivation; TM3, soybean intercropping cultivation.

An analysis was conducted on the dominant fungal genera with relative abundances greater than 1.5% among the 439 fungal genera in all samples across the three cultivation patterns. The results showed (Fig. 6B) that there were 11 dominant fungal genera with relative abundances greater than 1.5% in TM1, 10 in TM2, and 5 in TM3. The dominant fungal genera in TM1 were *Mortierella* (17.8%), *unidentified* (14.7%), *unclassified_Fungi* (8.4%), *Fusarium* (7.3%), *unclassified_Ascomycota* (6.4%), *Plectosphaerella* (2.7%), *Cladosporium* (2.3%), *Tetracladium* (2.1%), *unclassified_Basidiomycota* (2.0%), *Ceratobasidium* (2.0%), and *Rhizoctonia* (1.6%). Compared to TM1, TM2 shared 6 of these dominant genera, with *Mortierella* showing a relative abundance increase of 7.1% and *Fusarium* decreasing by 4.5%; the distinct dominant genera are *Mycofalcella* (2.4%), *unclassified_Saccharomycetales* (2.0%), *Tetracladium* (2.0%), and *Ramicandelaber* (1.7%). TM3 shared five dominant fungal genera compared with TM1; the relative abundance of *unidentified* and *unclassified_Fungi* escalated by 21.2% and 22.5%, respectively, while *Mortierella* and *unclassified_Ascomycota* witnessed reductions of 6.8% and 3.9%, respectively.

Subsequent analysis of some detrimental fungal genera (Table 4) revealed that the relative abundances of *Fusarium*, *Rhizoctonia*, *Ceratobasidium*, and *Plectosphaerella* in TM2 were reduced by 2.7-fold, 5.2-fold, 2.3-fold, and 1.8-fold, respectively, compared to TM1 (*P* < 0.05); similarly, in TM3, the relative abundances of the detrimental fungal genera *Fusarium*, *Stagonosporopsis*, *Rhizoctonia*, *Colletotrichum*, *Ceratobasidium*, and *Plectosphaerella* were reduced by 6.2-fold, 1.9-fold, 71.6-fold, 2.7-fold, 97-fold, and 3.22-fold, respectively, compared to TM1 (*P* < 0.05).

**Table 4:** Relative abundance of some detrimental fungal genera from different cultivation patterns.

Genus	TM1	TM2	TM3
*Fusarium* (%)	7.510 ± 1.191 a	2.824 ± 0.361 b	1.206 ± 0.352 c
*Stagonosporopsis* (%)	0.961 ± 0.111 a	0.836 ± 0.215 a	0.499 ± 0.142 b
*Rhizoctonia* (%)	1.576 ± 0.197 a	0.302 ± 0.052 b	0.022 ± 0.008 c
*Colletotrichum* (%)	0.919 ± 0.076 a	0.701 ± 0.077 ab	0.336 ± 0.062 c
*Ceratobasidium* (%)	2.037 ± 0.252 a	0.903 ± 0.154 b	0.021 ± 0.013 c
*Plectosphaerella* (%)	2.669 ± 0.442 a	1.473 ± 0.139 b	0.829 ± 0.063 c

**Notes:**

Different letters indicate significant differences at *P* < 0.05. Data are presented as the mean ± standard deviation (*n* = 3).

TM1, open-field cultivation; TM2, film-mulched cultivation; TM3, soybean intercropping cultivation.

### Analysis of differential taxa from different cultivation patterns

**Figure 7:**
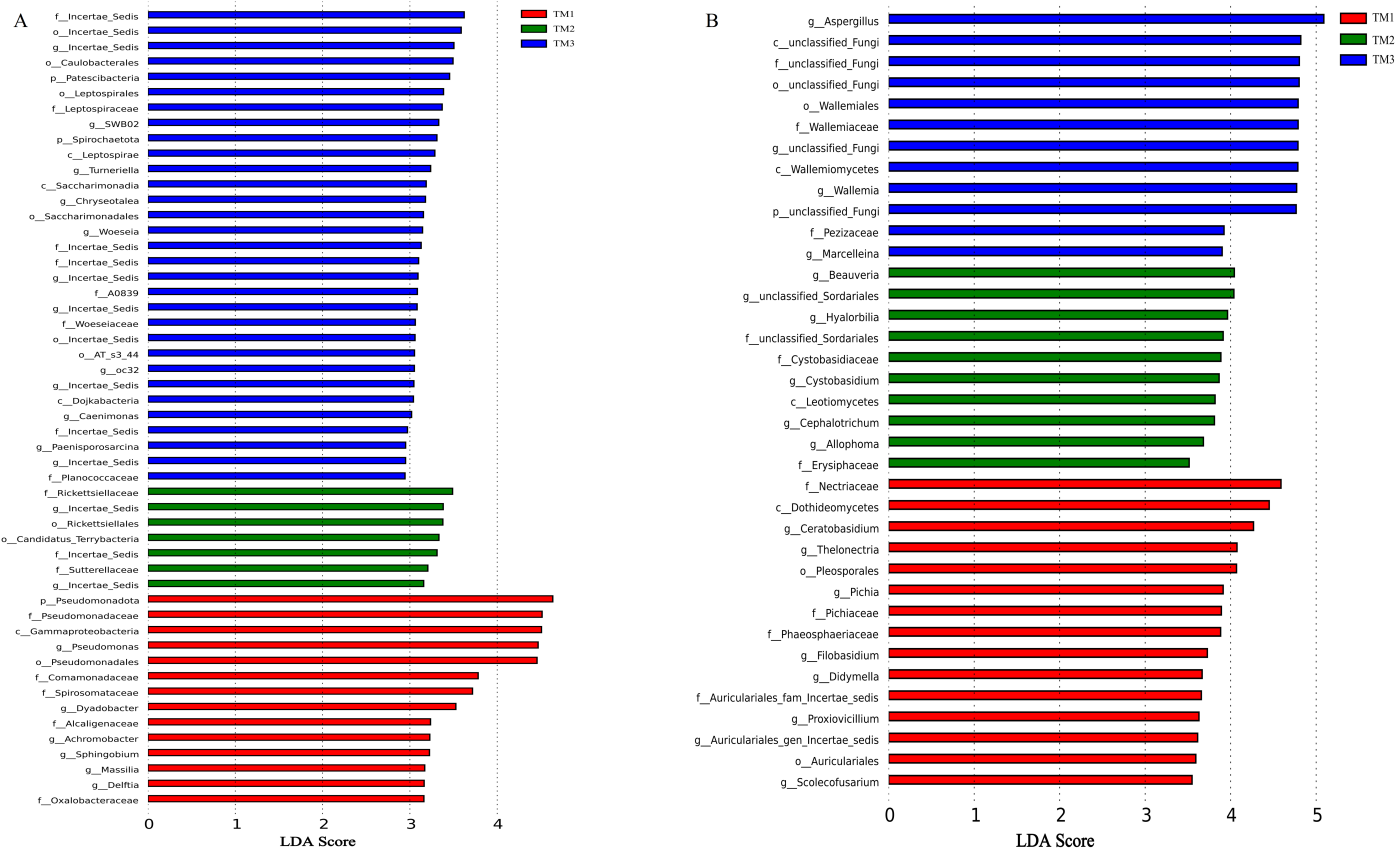
LEfSe analysis of rhizosphere soil microbial communities of *T. kirilowii* from different cultivation patterns. (A) Bacteria. (B) Fungi. TM1, open-field cultivation; TM2, film-mulched cultivation; TM3, soybean intercropping cultivation.

The influence of cultivation patterns on bacterial and fungal communities was clarified through LEfSe analysis (LDA score > 2.5, *P* < 0.05). The results showed that the cultivation pattern significantly affected the bacteria and fungi in the rhizosphere soil of *T. kirilowii* (Fig. 7). For bacteria, 14, 7, and 31 taxa were significantly enriched in TM1, TM2, and TM3, respectively. At the bacterial phylum level, Pseudomonadota was significantly enriched in TM1; Patescibacteria and Spirochaetota were significantly enriched in TM3. At the bacterial genus level, *Pseudomonas* and *Dyadobacter* were significantly enriched in TM1; *Incertae_sedis* was significantly enriched in TM2 and TM3 (Fig. 7A). For fungi, 15, 10, and 12 taxa were enriched in TM1, TM2, and TM3, respectively. At the fungal genus level, *Ceratobasidium* was significantly enriched in TM1, *Beauveria* was significantly enriched in TM2, and *Aspergillus* was significantly enriched in TM3 (Fig. 7B).

### Correlation analysis between microbial communities and soil nutrients

**Figure 8:**
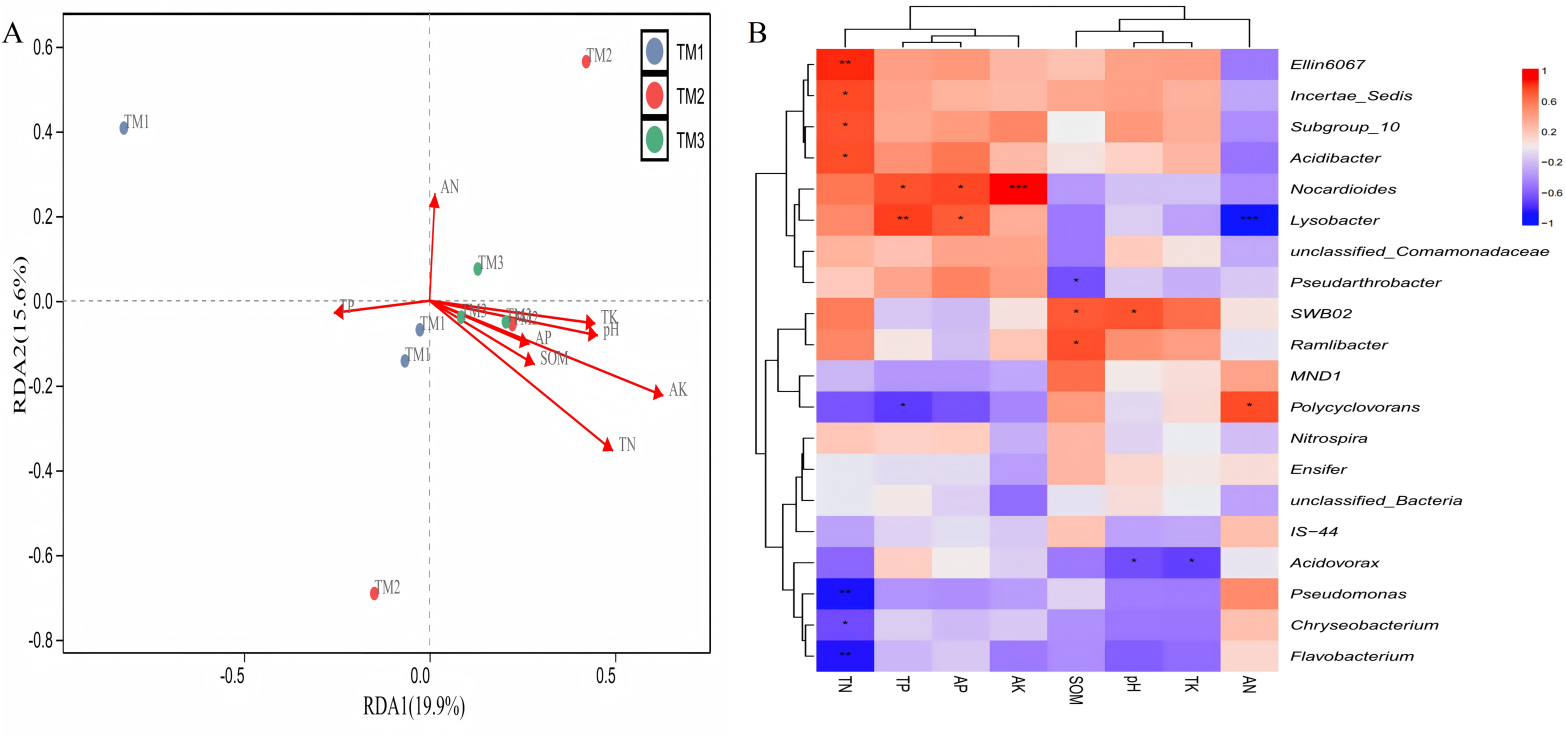
Correlation analysis between bacterial genera of *T. kirilowii* and soil nutrient indicators. (A) RDA analysis between various bacterial genera and soil nutrient indicators. TM1, open-field cultivation; TM2, film-mulched cultivation; TM3, soybean intercropping cultivation; SOM, organic matter; AN, available nitrogen; AP, available phosphorus; AK, available potassium; TN, total nitrogen; TP, total phosphorus; TK, total potassium. (B) Spearman correlation analysis of dominant bacterial genera and soil nutrient indicators. *, represents *P* < 0.05; **, represents *P* < 0.01; ***, represents *P* < 0.001.

Correlation between various bacterial genera and soil nutrients demonstrated (Fig. 8A) that the cumulative explained variation of the first axis (RDA1) and second axis (RDA2) of bacterial genera was 35.5%. Three cultivation patterns can be clearly distinguished in the figure, indicating that there were significant differences in soil bacterial communities among different cultivation patterns. TN (R = 0.459, *P* = 0.013) and AK (R = 0.637, *P* = 0.037) content significantly influenced the distribution of soil bacterial genera. Correlation analysis between dominant bacterial genera and soil nutrients revealed (Fig. 8B) that TN content was extremely significantly positively correlated with the relative abundance of *Ellin6067* and extremely significantly negatively correlated with *Pseudomonas* and *Flavobacterium*; TP content was extremely significantly positively correlated with the relative abundance of *Lysobacter*; AK content was extremely significantly positively correlated with the relative abundance of *Nocardioides*; and AN content was extremely significantly negatively correlated with the relative abundance of *Lysobacter*.

**Figure 9:**
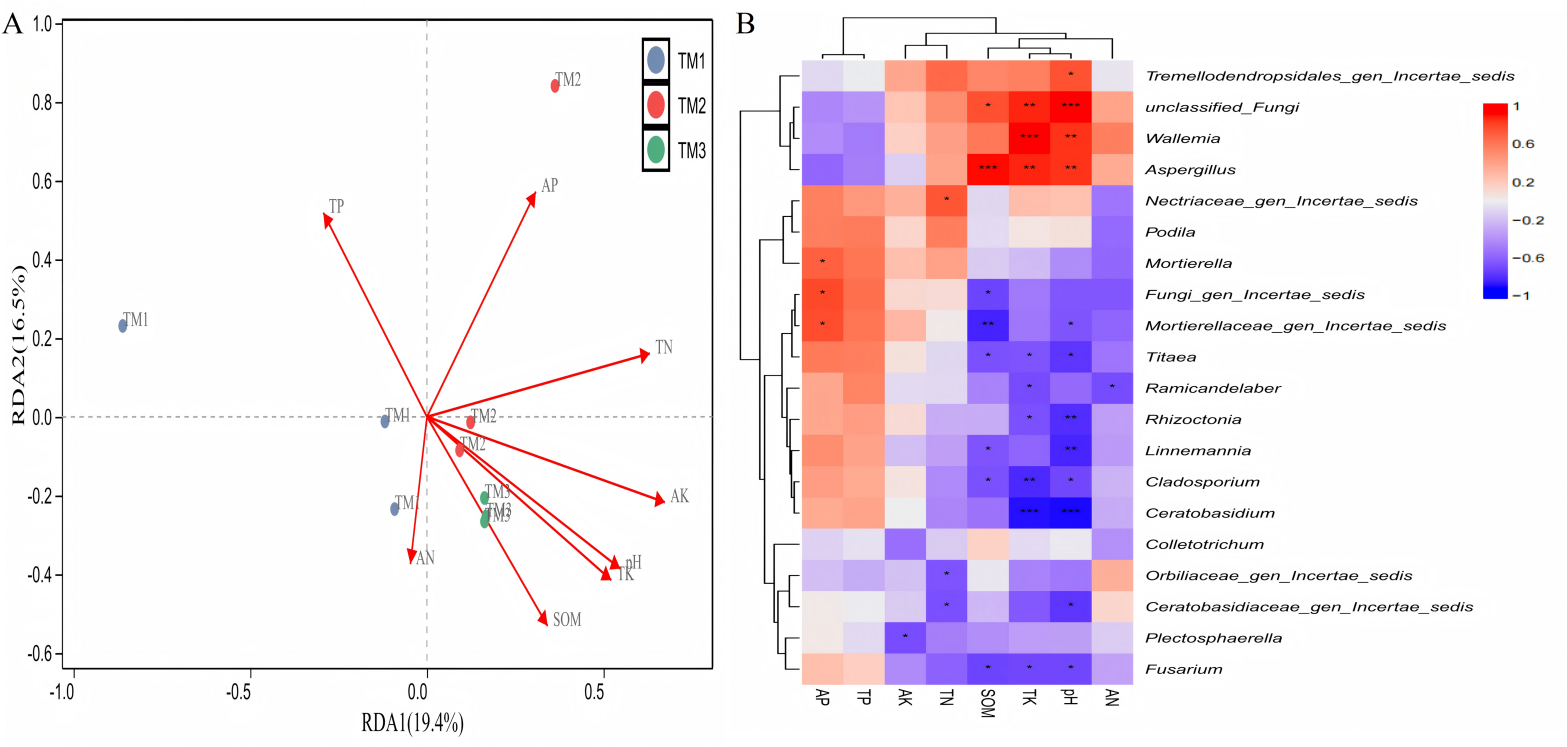
Correlation analysis between fungal genera of *T. kirilowii* and soil nutrient indicators. (A) RDA analysis between various fungal genera and soil nutrient indicators. TM1, open-field cultivation; TM2, film-mulched cultivation; TM3, soybean intercropping cultivation; SOM, organic matter; AN, available nitrogen; AP, available phosphorus; AK, available potassium; TN, total nitrogen; TP, total phosphorus; TK, total potassium. (B) Spearman correlation analysis of dominant fungal genera and soil nutrient indicators. *, represents *P* < 0.05; **, represents *P* < 0.01; ***, represents *P* < 0.001.

The analysis of the correlation between various fungal genera and soil nutrients (Fig. 9A) revealed that the cumulative explained variance of the first axis (RDA1) and second axis (RDA2) of fungal genera was 35.9%. Three cultivation patterns were clearly distinguishable in the figure, indicating that there were significant differences in soil fungal communities among different cultivation patterns. TP (R = 0.801, *P* = 0.011), TK (R = 0.882, *P* = 0.001), SOM (R = 0.697, *P* = 0.008) content, and pH (R = 0.849, *P* = 0.001) significantly or extremely significantly influenced the distribution of soil fungal genera. Correlation analysis between dominant fungal genera and soil nutrients revealed (Fig. 9B) that SOM content was extremely significantly positively correlated with the relative abundance of *Aspergillus* and extremely significantly negatively correlated with *Mortierellaceae_gen_Incertae_sedis*; TK content was extremely significantly positively correlated with the relative abundance of *unclassified_Fungi*, *Wallemia*, and *Aspergillus*, and extremely significantly negatively correlated with the relative abundance of *Cladosporium* and *Ceratobasidium*; pH was extremely significantly positively correlated with the relative abundance of *unclassified_Fungi*, *Wallemia*, and *Aspergillus*, and extremely significantly negatively correlated with the relative abundance of *Rhizoctonia*, *Linnemannia*, and *Ceratobasidium*.

## Discussion

### Effects of different cultivation patterns on rhizosphere soil microbial diversity of *T. kirilowii*

Soil microorganisms are central to maintaining soil health and are highly sensitive to environmental changes, capable of rapidly reflecting changes in soil quality (Dubey et al., 2019). As such, the structure and diversity of soil microbial communities are frequently employed as crucial indicators for assessing soil health (Jiao et al., 2022). Soil microorganisms can provide crops with various resources, and their community structure and diversity can influence aboveground ecosystems, thereby affecting crop productivity (Romero et al., 2023). Therefore, investigating the effects of cultivation patterns on the structure and diversity of soil microbial communities in the rhizosphere of *T. kirilowii* can provide deeper insights into soil quality changes and foster the sustainable and judicious use of soil ecosystems. The results of this study indicate that after film-mulched cultivation and soybean intercropping cultivation, the number of bacterial OTUs increased, while the number of fungal OTUs decreased. The cultivation pattern has altered the community composition of soil microorganisms in the rhizosphere, which is similar to the results reported by Li et al. (2021b) in corn cultivation and Zhao et al. (2022) in corn/soybean intercropping. Soil microbial abundance indices and diversity indices are frequently employed as indicators to characterize community diversity. Crops exhibit selectivity in the aggregation of rhizosphere microbial communities, which can influence changes in soil microbial structure through differences in root exudates (Chen et al., 2022). Crops in intercropping systems modify soil microenvironments *via* root exudate-mediated competition for soil nutrients, consequently influencing soil microbial diversity (Wang et al., 2018). Soybean-maize intercropping boosts bacterial and fungal diversity, changes soil community structure *via* interspecific interactions, and improves the relative abundance of beneficial bacterial taxa involved in nutrient cycling (Guo et al., 2024). Root exudates and soil physiochemical characteristics were shown to be substantially linked with microbial community shifts in the rhizosphere across rhizobial treatments (Liu et al., 2021b). This research reveals that soybean intercropping cultivation promoted the accumulation of soil nutrients, significantly enriched the biomarker *Aspergillus*, and markedly altered the microbial community structure and diversity in the rhizosphere soil of *T. kirilowii*. This is similar to the findings of Zhao et al. (2022) in corn/soybean intercropping and Xiao et al. (2016) in *Panax ginseng* cultivation. This possibly can be attributed to the increased quantity and diversity of *T. kirilowii* root exudates in soybean intercropping cultivation, providing richer energy sources for rhizosphere microorganisms and thereby enhancing bacterial diversity. Concurrently, interactions between *T. kirilowii* and soybean, along with the presence of rhizobia, may generate specific root exudates that lead to the formation of rhizosphere microbial communities adapted to these exudates, thereby altering the overall metabolic activity of soil microorganisms and promoting the formation of diverse microbial community structures. Although many studies have reported that film-mulched cultivation and intercropping cultivation enhance rhizosphere fungi diversity (Dang et al., 2020; Kang et al., 2020; Li et al., 2022b), this study discovered that film-mulched cultivation and intercropping cultivation significantly reduced the fungi diversity in the rhizosphere soil of *T. kirilowii* compared to the open-field cultivation, with a significant decrease in the abundance and diversity of some detrimental fungal species. This may be related to the improved soil environment for *T. kirilowii* growth resulting from film-mulched cultivation and soybean intercropping cultivation.

### Effects of different cultivation patterns on the rhizosphere soil microbial structure of *T. kirilowii*

Bacteria and fungi are important components of soil microorganisms, with bacteria playing an extremely important role in crop growth and development, pest and disease control, material formation and decomposition, soil nutrient cycling, fertility maintenance, and ecological environment improvement (Cao et al., 2017). This study demonstrates that Pseudomonadota, Acidobacteriota, unclassified_Bacteria, Bacteroidota, and Chloroflexota are the dominant bacterial phyla in the rhizosphere soil of *T. kirilowii*, with Pseudomonadota having the highest relative abundance (28.8–34.7%) and Acidobacteriota having a relative abundance of 17.5–20.6%. Pseudomonadota has been found to be enriched in the rhizosphere of various plants (Li et al., 2021a; Naumova et al., 2022), indicating its strong adaptability. Acidobacteriota can degrade lignin and cellulose to provide nutrients for the soil. This study showed that the relative abundance of Acidobacteriota in TM2 and TM3 increased by 8.8% and 3.1%, respectively, compared to TM1, indicating that film-mulched cultivation and soybean intercropping cultivation promote the enrichment of Acidobacteriota, thereby influencing soil nutrients. Furthermore, cropping patterns influence the relative abundance of bacterial phyla such as Bacteroidota and Chloroflexota, which play important roles in nutrient metabolism and energy production through photosynthesis. This study showed that the relative abundance of Bacteroidota in TM2 and TM3 increased by 2.47% and 1.71%, respectively, compared to TM1, while the relative abundance of Chloroflexota in TM3 increased by 4.23% compared to TM1. These findings align with those of Xu et al. (2022) on pea/corn intercropping. Beneficial rhizosphere microorganisms play a crucial role in promoting plant growth and development, helping plants resist foreign invasions through nutrient competition, participating in soil nutrient cycling, and inducing plant resistance (Bhat et al., 2022). *Nitrosospira* possesses ammonia-oxidizing activity (Rice et al., 2016), *Bryobacter* can degrade cellulose and lignin and has biocontrol functions (Liu et al., 2020); *Steroidobacteraceae* promotes organic matter degradation and carbon-nitrogen cycling (Xie et al., 2025), while *Pseudarthrobacter* and *Agromyces* can regulate carbon cycling (Cheng et al., 2024), and *Nocardioides* plays an important role in environmental adaptation (Zhang et al., 2024a). The results of this study indicate that cultivation patterns alter the relative abundance of beneficial microorganisms in the rhizosphere soil of *T. kirilowii*. The relative abundance of beneficial bacterial genera such as *Nitrosospira*, *Bryobacter*, *Pseudarthrobacter*, *unclassified_Steroidobacteraceae*, *Nocardioides*, and *Agromyces* in film-mulched cultivation was significantly higher than that in open-field cultivation. Furthermore, the relative abundance of beneficial bacterial genera, including *Pseudarthrobacter*, *unclassified_Steroidobacteraceae*, and *Nocardioides* in soybean intercropping cultivation also significantly surpasses that in open-field cultivation. This result is similar to the findings of Kang et al. (2020) in potato cultivation.

Soil fungi contribute to the energy flow and material cycling within terrestrial ecosystems, ensuring their proper function. Acidobacteriota can promote organic matter degradation and cause crop diseases (Lu et al., 2023), while Mortierellomycota have functions such as promoting the decomposition of organic matter, nutrient cycling, and enhancing crop immunity (Sun et al., 2024). The results of this study indicate that Acidobacteriota and Mortierellomycota are the dominant phyla in the rhizosphere soil fungal communities of *T. kirilowii*. However, the cultivation patterns alter the relative abundance of Acidobacteriota and Mortierellomycota. Compared to open-field cultivation, film-mulched cultivation increased the relative abundance of Mortierellomycota by 8.0%, while the relative abundance of Acidobacteriota decreased by 5.3%. Compared with open-field cultivation, the relative abundances of Acidobacteriota and Mortierellomycota in soybean intercropping cultivation decreased by 5.4% and 6.2%, respectively. These results suggest that film-mulched cultivation and soybean intercropping cultivation can mitigate the risk of disease outbreaks. The detrimental fungal genera *Ceratobasidium and Fusarium* can cause wilt disease and root rot in crops (Li et al., 2025; Husna, Zakaria & Mohamed, 2020), while *Stagonosporopsis* is associated with gummy stem blight disease in Cucurbitaceae plants and wilt disease in Asteraceae plants (Jeong et al., 2022; Vaghefi et al., 2016), *Rhizoctonia* is closely associated with tobacco leaf spot and root rot (Gonzalez et al., 2011), *Colletotrichum* causes anthracnose in crops (Choi, Shin & Choi, 2024), and *Plectosphaerella* is closely related to root rot in crops (Elmer et al., 2019). The results of this study indicate that film-mulched cultivation and soybean intercropping cultivation significantly reduce the relative abundance of some detrimental fungal genera. The biomarker *Ceratobasidium* was significantly enriched in open-field cultivation, which was markedly decreased by 2.3-fold and 97-fold in film-mulched cultivation and soybean intercropping cultivation, respectively. The relative abundance of detrimental fungal genera such as *Fusarium*, *Rhizoctonia*, and *Plectosphaerella* in film-mulched cultivation was reduced by 2.7-fold, 5.2-fold, and 1.8-fold, respectively, compared to open-field cultivation (*P* < 0.05); in soybean intercropping cultivation, the relative abundances of the detrimental fungal genera *Fusarium*, *Stagonosporopsis*, *Rhizoctonia*, *Colletotrichum*,, and *Plectosphaerella* were reduced by 6.2-fold, 1.9-fold, 71.6-fold, 2.7-fold, and 3.22-fold, respectively, compared to open-field cultivation (*P* < 0.05). The above results indicate that film-mulched cultivation and soybean intercropping cultivation influence the abundance and structure of bacteria and fungi in the soil, increasing the relative abundance of some beneficial bacterial genera and decreasing the relative abundance of some fungal genera that cause severe diseases. This improves the microbial environment for *T. kirilowii* growth and reduces the risk of disease occurrence in *T. kirilowii*.

### Effects of different cultivation patterns on the rhizosphere soil nutrients and microbial communities of *T. kirilowii*

Plant type, soil type, and cultivation patterns significantly influence the structure and diversity of soil microbial communities. Soil organic matter and soil nutrients are the primary internal factors affecting the structure of soil microbial communities (Song et al., 2023; Tong et al., 2021). This study indicates that cultivation patterns influence soil nutrients. The content of AK, TN, TP, and the pH value of the rhizosphere soil in film-mulched cultivation and soybean intercropping cultivation were significantly higher than those in open-field cultivation, suggesting that film-mulched cultivation and soybean intercropping cultivation promote soil nutrient accumulation to some extent. This is consistent with the findings of Liao et al. (2024). Soil nutrients influence the community structure of rhizosphere microorganisms, thereby impacting crop growth. Zhang et al. (2024d) found that soil bacterial communities are significantly correlated with soil pH, AK, AP, and TN in morifolium-maize intercropping, while soil fungal communities are significantly correlated with pH and AK. Chen et al. (2025a) revealed that pH, AP, and AK are critical determinants of bacterial community changes in Yunnan bamboo shoots, while pH, SOM, AK, and TN predominantly influenced fungal community changes. This study indicates that TN and AK content significantly influence the distribution of bacterial genera in soil, while TP, TK, SOM content, and pH value significantly or extremely significantly influence the distribution of fungal genera in soil, clarifying the impact of soil nutrients on changes in the microbial community of the *T. kirilowii* rhizosphere soil. Related studies have shown that soil nutrients are closely related to the species and abundance of soil microbial communities (Naumova et al., 2022; Zhang et al., 2024d). The results of this study show that AK content is extremely significantly positively correlated with the relative abundance of *Nocardioides*, TK content is extremely significantly negatively correlated with the relative abundance of *Ceratobasidium*, and pH is extremely significantly negatively correlated with the relative abundance of *Rhizoctonia* and *Ceratobasidium*. These findings are similar to the results of Zhang et al. (2024c) on sweet potatoes. The results above all suggest that in agricultural production, soil nutrients can be regulated to increase the abundance of beneficial bacteria and reduce the abundance of detrimental fungi in the soil, providing a reference for effectively mitigating the continuous cropping barriers of *T. kirilowii*.

## Conclusions

Cultivation patterns reshape the rhizosphere micro-ecosystem of *T. kirilowii*. Film-mulched cultivation and soybean intercropping cultivation improved soil nutrients, increased bacterial community diversity and the relative abundance of beneficial bacterial genera, and decreased the relative abundance of detrimental fungal genera. The results demonstrate that cultivation patterns can effectively mitigate the continuous cropping barriers by optimizing the soil nutrient environment and fostering a disease-suppressive soil microbiome. These findings provide a scientific basis for recommending film-mulched cultivation and soybean intercropping cultivation as sustainable cultivation strategies in *T. kirilowii* production. Despite the above achievements of this study, there are also some limitations, including the limitations inherent to metabarcoding-based analyses and lack of functional microbial information. In future studies we will employ metagenomics and transcriptomics approaches to elucidate the functional pathways underlying these microbial changes and thus formulate targeted microbiome management strategies for *T. kirilowii* cultivation.

## Supplemental Information

10.7717/peerj.20459/supp-1 Supplemental Information 1 Nutrient indicators in the rhizosphere soil of *T.kirilowii* from different cultivation patterns.

10.7717/peerj.20459/supp-2 Supplemental Information 2 Relative abundance of some important functional bacterial phyla, beneficial bacterial genera, and detrimental fungal genera from different cultivation patterns.

## Additional Information and Declarations

### DNA Deposition

The following information was supplied regarding the deposition of DNA sequences:

The sequence data are available in the NCBI Sequence Read Archive: SRR35124295–SRR35124303.

### Data Availability

The following information was supplied regarding data availability:

Data are available in the NCBI Sequence Read Archive: SRR35124295–SRR35124303.
